# The effect of metallic Fe(ii) and nonmetallic S codoping on the photocatalytic performance of graphitic carbon nitride

**DOI:** 10.1039/c8ra00056e

**Published:** 2018-02-16

**Authors:** Hailong Dou, Shaohui Zheng, Yongping Zhang

**Affiliations:** Faculty of Materials and Energy, Southwest University Chongqing 400715 China zhangyyping@yahoo.com

## Abstract

The metallic Fe(ii) ion and nonmetallic S codoped g-C_3_N_4_ photocatalyst was synthesized through the polymerization of melamine, ferrous chloride and trithiocyanuric acid (TCA) at elevated temperature. The performance of Fe(ii)–S codoped g-C_3_N_4_ compounds in RhB photocatalytic degradation was found to increase 5 times. This significant enhancement in catalytic activity is probably related to the enhanced visible light adsorption and the mobility of photoinduced electron/hole pairs, attributable to bandgap narrowing and also lowering in the surface electrostatic potential compared to that of the pure g-C_3_N_4_ nanosheets. XRD and XPS results indicate that the Fe species binds with N-atoms to form Fe–N bonds in the state of Fe(ii) ions. Fe(ii) doping increases the specific surface area, and enhances the photoinduced electron/hole pairs illustrated by PL, EIS spectra and transient photocurrent response measurements. The theoretical results show that divalent Fe(ii) ions coordinating in the pore centre among three triazine units form discrete dopant bands and S dopants substituting the N in triazine skeletons excite much stronger delocalized HOMO and LUMO states, facilitating the migration of photogenerated charge carriers, thus enhancing the visible-light driven photocatalytic performance.

## Abstract 2

The photoinduced electrons jump more easily to the conduction band of g-C_3_N_4_ for the Fe impurity band locates above the valence band acting a bridge for electron transfer.

## Graphical abstract

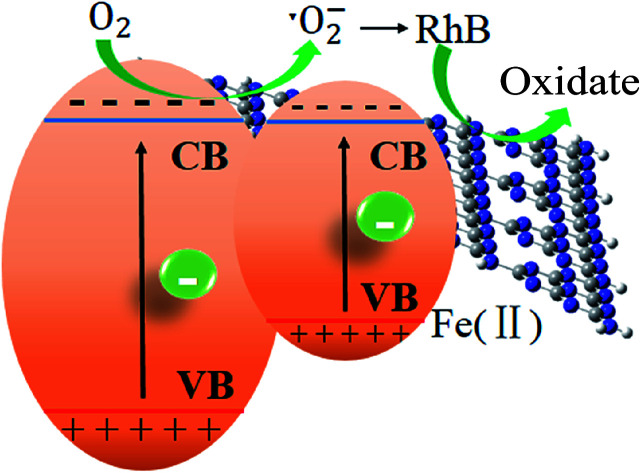

## 1. Introduction

Pollutant degradation and clean energy generation through semiconductor photocatalysis have been important research topics. Photocatalysis provides certain promising approaches in water splitting for H_2_ evolution and pollutant degradation using solar energy directly and aroused considerable interest from research experts in materials science and chemistry. It is a green technology to decompose pollutants into innocuous molecules *via* photocatalysis, without secondary pollution. However, there are still several challenges, including low quantum efficiency and insufficient visible light adsorption of photocatalysts.

The visible light photocatalysts based on graphene have drawn great attention due to their good absorption performance, excellent electrical conductivity and high specific surface area.^1–4^ However, the application of 2D graphene is hindered by its zero band gap. The graphitic carbon nitride (g-C_3_N_4_) has layered structure similar to graphene and possesses an appropriate band structure and bandgap of 2.7 eV allowing it to serve as a visible light driven photocatalyst for solar energy conversion. It was found to have potential applications in water splitting to generate H_2_, pollutant degradation and CO_2_ reduction.^5–8^ The major issue related to g-C_3_N_4_ is the rapid combination of the photoinduced charge carriers, resulting in a low quantum efficiency of photocatalytic reactions.

Great endeavors have been made to improve the performance of g-C_3_N_4_ ever since its emergence as a photocatalyst. One strategy was to fabricate nano/mesoporous structures with a soft or hard template,^9–12^ further increasing the specific surface area, and thus improving the photon absorption in the visible light region. Another was to engineer the band structures of g-C_3_N_4_ catalysts to separate the electron/hole pair effectively by coupling with metal particles,^13–15^ doping metal or non-metal elements,^16–20^ and forming heterojunction with other semiconductors, such as ZnO, CuO, TiO_2_ and CdS,^21–24^*etc.* The studies of doping elements in g-C_3_N_4_ revealed that anion ion doping tunes the conduction band through the hybridization of p-orbitals of doped element with the p-orbitals of matrix carbon nitride, while cations adjusts the valence band by generating a discrete band *via* 3d orbitals of transition metal elements. Anion and cation codoping may enable us to modify the conduction and valence bands simultaneously, thus tuning the bandgap. Meanwhile, anion and cation codoping maintains the charge balance in the g-C_3_N_4_ nanosheets, and stabilizes its photocatalytic performance. In practice, complexity occurs in specific cases related to different doping sites and variation of valence state of transition metal elements. Therefore, further experimental and theoretical investigations are essential to develop high performance catalysts and gain deeper insight into the mechanism of the enhanced photocatalytic performance *via* metal and nonmetal element codoping.

In this paper, we synthesized a Fe(ii) and S codoped g-C_3_N_4_ photocatalyst by calcifying melamine, ferrous chloride and trithiocyanuric acid and expatiated its mechanism by intertwining experimental observation and theoretical calculation. The experimental results reveal that the Fe(ii)–S codoped g-C_3_N_4_ exhibits superior photodegradation for RhB under visible light irradiation. Density functional theory (DFT) calculations demonstrate that the Fe(ii) and S doping reduces the bandgap and increases the reactive sites, facilitating the transfer of photogenerated electron–hole pairs.

## 2. Experimental details

### 2.1 Synthesis of the pure, Fe(ii) doped, Fe(ii)–S codoped g-C_3_N_4_

All chemicals used herein were analytical grade and used without further purification. Melamine (C_3_H_6_N_6_, ≥99.5%), ferrous chloride (Fe(ii)Cl_2_, ≥99%), and trithiocyanuric (C_3_H_3_N_3_S_3_, ≥95%) were supplied by Sinopharm Chemical Reagent Co. Ltd.

In the typical experiment, iron(ii) chloride (Fe(ii)Cl_2_, 0.13 g) was dissolved into 20 mL 3 M nitric acid. Then the solution was fully mixed with 3 g melamine (dissolved in 60 mL methanol solution with 50% water) inside a beaker and dried at 60 °C overnight. The resulting white substance of 2.52 g was mixed with 1.77 g trithiocyanuric acid (TCA). The mixture was ground into powders, transferred to a silica crucible with cover, and then heated at 550 °C for 2 h under nitrogen environment. The resulting yellow powders were ground to produce Fe(ii)–S codoped g-C_3_N_4_ samples (denoted as Fe(ii)/S–g-CN). The Fe(ii) doped g-C_3_N_4_ (Fe(ii)–g-CN) was prepared in the same way without TCA involved in the preparation process. The pure g-C_3_N_4_ (g-CN) was synthesized by thermally heating melamine (M) powder (2.56 g) at 550 °C for 2 h under nitrogen atmosphere.

### 2.2 Characterization

Surface morphologies of the prepared samples were investigated using a scanning electron microscope (SEM, JSE-7800F, Jeol). Their crystalline structures were characterized by X-ray diffraction (XRD) patterns obtained using Shimadzu XRD7000 with Cu Kα radiation (*λ* = 1.5418 Å). Fourier transform infrared spectroscopic study (FTIR) was conducted using a Perkin Elmer spectrometer in KBr pellets. The composition and chemical state of the elements in the catalysts were measured using an X-ray photoelectron spectrometer (XPS, VG ESCALAB 250) with Al Kα radiation (*hν* = 1486.8 eV). Ultraviolet-visible (UV-vis) absorption spectra were obtained on U-3310 spectrometer (Hitachi, Japan) in the wavelength range of 300 to 800 nm. Photoluminescence (PL) spectroscopic investigations were carried out on a F-7000 fluorescence spectrophotometer (Hitachi, Japan) with an excitation wavelength at 273 nm using a 150 W Xe lamp as the excitation source. The Brunauer–Emmett–Teller (BET) surface area was measured with an ASAP-2010 analyzer. The photocurrent measurements were conducted on an electrochemical workstation in a standard three-electrode system, using a platinum wire and the saturated Ag/AgCl electrode as the counter electrode and reference electrode, respectively. The working electrode was prepared by coating the catalysts on a 1.8 cm × 1.2 cm fluorine-doped tin oxide (FTO) glass substrate. A 300 W Xe lamp with a 420 nm cut-off filter was used as a light source.

### 2.3 Photodegradation of RhB

The photocatalytic performance was examined by monitoring the degradation of Rhodamine B (RhB) in an aqueous solution at room temperature under visible light irradiation using a 300 W xenon lamp with 400 nm cutoff filters as light source. In each experiment, 10 mg of photocatalyst was dispersed in 50 mL RhB aqueous solution with an initial concentration of 10 mg l^−1^. Prior to irradiation, the suspension was magnetically stirred in the dark room for 60 min to reach sorption equilibrium. During the photocatalysis process, 1 ml of the sample was withdrawn from the reaction cell at 10 min intervals and then centrifuged for measuring the characteristic UV-vis absorption of RhB. The absorption peak maximum was employed in evaluating the concentration of RhB. The degradation rate of RhB can be calculated accordingly:Degradation rate = (*C*_0_ − *C*_*t*_)/*C*_0_where *C*_0_ is the sorption equilibrium concentration of RhB and *C*_*t*_ is the concentration of RhB at reaction time *t*.

## 3. Results and discussion

**Fig. 1:**
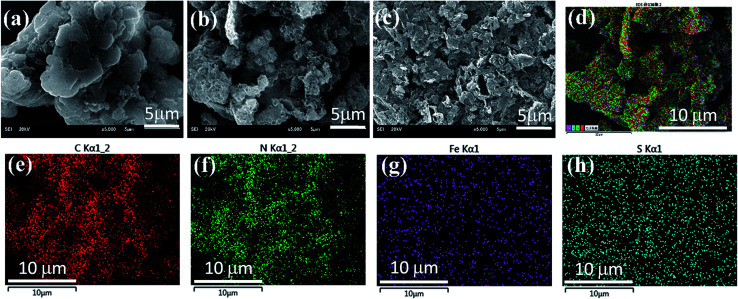
SEM images of (a) the pure, (b) Fe(ii)/S and (c) Fe(ii) doped g-C_3_N_4_ photocatalysts. Elemental EDX overlapped images of C, N, Fe and S (d) and mappings for C (e), N (f), Fe (g) and S (h) for the Fe(ii)/S codoped g-C_3_N_4_.

The morphologies of the representative samples are shown in SEM micrographs. Fig. 1(a) indicates that the pure g-C_3_N_4_ has a layered structure with some crinkled flakes. These g-C_3_N_4_ layers heaped together to form irregular particles. The Fe(ii) and S codoped g-C_3_N_4_ sheets in Fig. 1(b) become the lamella structures curved even more than that of the Fe(ii) doped g-C_3_N_4_ (Fig. 1(c)) with more irregular porous structures. The mesoporous structures may be caused by the decomposition of TCA during polymerization process, while the crimped structures are arisen from the larger radii of doped Fe(ii) and S than those of the host C and N atoms.^25^ The Fe(ii) + S codoped g-C_3_N_4_ has a large number of mesopores, which demonstrates a higher specific surface area, possibly leading to easy transportation of pollutant to the interior surface *via* the interconnected mesopores. The energy dispersive X-ray (EDX) analysis shows that the composition of the Fe(ii)–S codoped g-C_3_N_4_ comprising rich carbon (C) and nitrogen (N) with dispersed Fe and S elements. The overlapped C, N, Fe and S element EDS image (Fig. 1(d)) suggests that the Fe(ii) and S elements are dispersed in g-C_3_N_4_ sheets. The EDX elemental mappings in Fig. 1(e–h) clearly show that the Fe and S elements are distributed homogeneously on the continuous C, N elemental background.

**Fig. 2:**
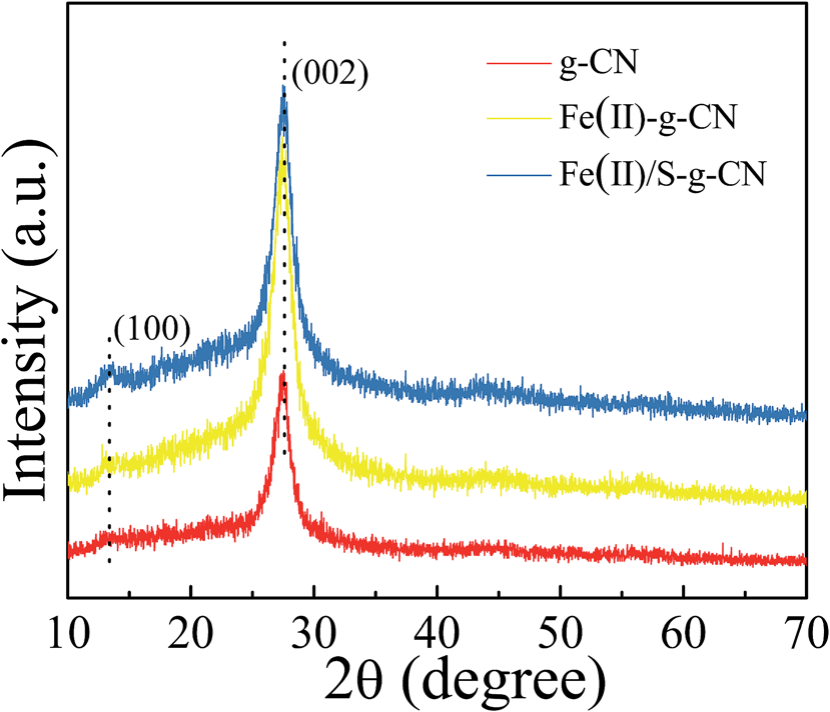
XRD patterns of the g-C_3_N_4_, Fe(ii) doped and Fe(ii)/S codoped of g-C_3_N_4_ catalysts.

The crystal structures of the pure, the Fe(ii)-doped and the Fe(ii)–S codoped g-C_3_N_4_ are demonstrated by their XRD patterns in Fig. 2. For the pure g-C_3_N_4_, XRD pattern displays two distinct diffraction peaks located at 2*θ* of about 13.1° and 27.3°, which are in good accordance with the characteristic (100) and (002) planes of g-C_3_N_4_.^24^ The peak for (100) plane corresponds to the repeating in-plane structural packing motif of the tri-*s*-triazine unit with a period of 0.67 nm, which is slightly smaller than the theoretical value of 0.73 nm. The smaller in-plane packing motif suggests that the structure of g-C_3_N_4_ layer is corrugated slightly after polymerization of melamine. The peak for (002) crystal plane of g-C_3_N_4_ is attributed to the lattice planes formed by stacking the conjugated aromatic systems into layered structure with an interlayer distance of 0.33 nm. This illustrates that the g-C_3_N_4_ has a layered structure similar to graphite. Upon Fe(ii) and S doping, the positions of these two peaks do not change. This result indicates that the Fe(ii) and S atoms are incorporated in the g-C_3_N_4_ layer, and light doping of Fe(ii) and S has no significant effect on the crystal structure of g-C_3_N_4_. The intensities of the diffraction peaks increase after Fe(ii) and S doing, indicating a better crystallinity than that of the pure g-C_3_N_4_.

**Fig. 3:**
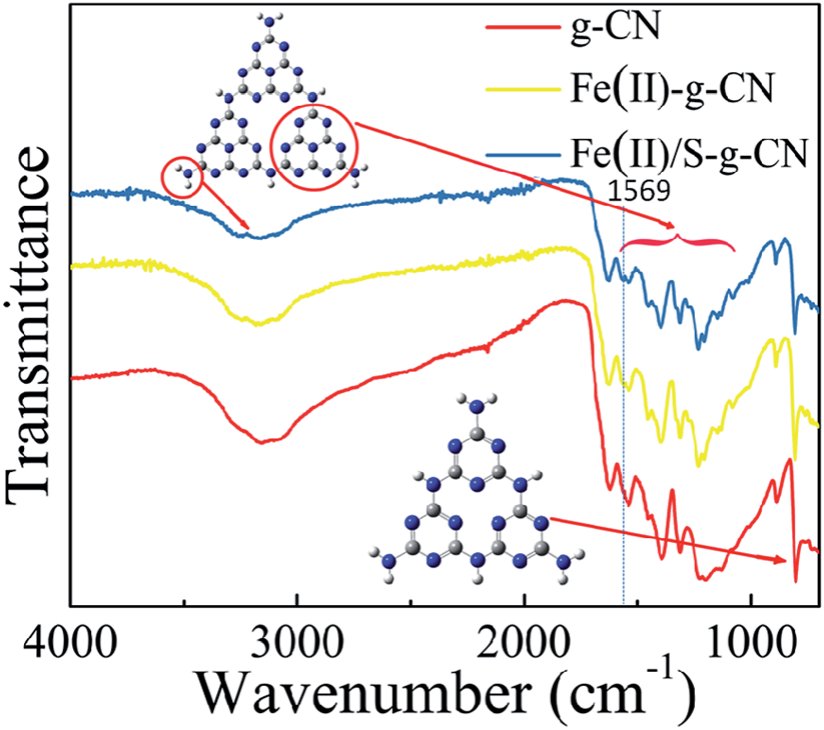
FT-IR spectra of the pure, Fe(ii) doped and Fe(ii)/S codoped of g-C_3_N_4_.

The FTIR spectra of the pure, the Fe(ii)-doped and the Fe(ii)–S codoped g-C_3_N_4_ catalysts are depicted in Fig. 3, in which the typical characteristic peaks at 1312, 1390, 1534, and 1625 cm^−1^ can be assigned to the stretching modes of the aromatic C–N heterocycle and peak at 805 cm^−1^ is ascribed to the breathing mode of the triazine units.^25–28^ The peak at 1228 cm^−1^ represents the stretching vibration of C–NH–C bridges.^28^ This demonstrates that the original graphitic C–N network in the structure of g-C_3_N_4_ is kept intact upon doping. The broad peaks at 3000–3400 cm^−1^ are ascribed to the stretching modes of N–H, indicating there exist N–H bonds at the edge of the polymerized triazine. Compared to the pure g-C_3_N_4_, a new peak at 1569 cm^−1^ appears in FTIR spectra for the Fe(ii)–S codoped g-C_3_N_4_, which can be attributed to the stretching mode of Fe–N bond in the coordination complex, confirming that the Fe ion coordinates at the pore site of g-C_3_N_4_ lattice.^26^ It is noteworthy that the FTIR spectrum does not change dramatically after Fe(ii) doping, which may show that the Fe(ii) ion has little interaction with the aromatic C–N rings.

**Fig. 4:**
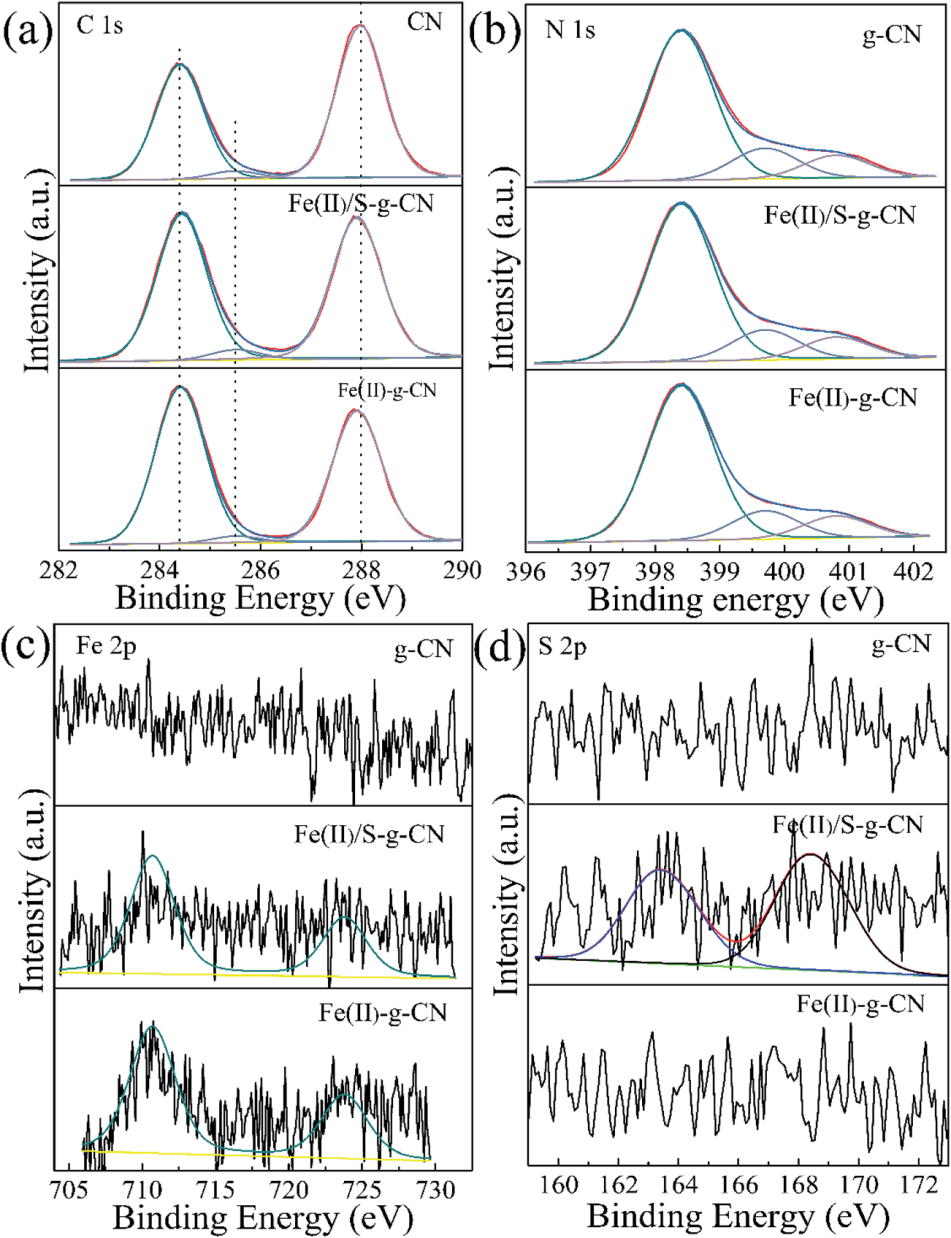
XPS high resolution spectra for C 1s (a) and N 1s (b), Fe 2p (c) and S 2p (d) of the pure g-C_3_N_4_ nanosheets (g-CN) and Fe(ii) doped g-C_3_N_4_ (Fe(ii)–g-CN) and Fe(ii) + S codoped g-C_3_N_4_ (Fe(ii)/S–g-CN).

Fig. 4 shows the high resolution XPS spectra of C, N, Fe and S elements in the pure g-C_3_N_4_, Fe(ii)-doped, and Fe(ii)–S codoped g-C_3_N_4_. The C 1s spectra of all three samples are shown in Fig. 4(a). For the pure g-C_3_N_4_ nanosheets, the peaks centered at about 287.98 eV (C1) is typically attributed to the sp^2^ hybrid C atoms bonded to N-containing aromatic skeleton rings (N–C=N). The peak at 285.48 eV (C2) is related to the C–NH_2_ bonded in the triazine ring of the reactant intermediary product.^10,18^ The peak at 284.40 eV (C3) could be attributed to the graphitic carbon (C–C and C=C).^28^ The binding energy of C1 shifts to a lower value of 287.90 eV after ferrous ion doping. This may be caused by the formation of Fe–N coordination bond leading to the partial breakage of the aromatic π bond to N–C–N, coexisting of N–C–N and N–C=N.^26^ The N 1s spectra for the pure g-C_3_N_4_, and the Fe(ii)-doped, Fe(ii)–S codoped g-C_3_N_4_ in Fig. 4(b) can be decomposed into three typical peaks located at about 398.40 eV (N1), 399.70 eV (N2), 400.80 eV (N3), which could be attributed to the sp^2^-hybridized aromatic N atoms bonded to carbon atoms (C–N=C), and sp^3^-hybridized N atoms of N(–C)_3_ and terminal amino functions (C–NH_2_), respectively.^29–33^ The S atomic dopant does not change the binding energy of C 1s spectrum since the element S has a similar electronegativity as the C atom.

The Fe 2p spectra for the Fe(ii)-doped, Fe(ii)–S codoped g-C_3_N_4_ in Fig. 4(c) show the peaks at 710.7 eV and 723.8 eV are ascribed to the splitting orbits of Fe 2p_3/2_ and 2p_1/2_, consistent with reported Fe^2+^ binding energies.^26^ This observation demonstrates that Fe(ii) ion forms coordination bond with the edge N atoms of hepazine.^31,33^ The Fe(ii) atom is imbedded in the pore centre among three tri-*s*-triazine units of g-C_3_N_4_ in the oxidation state of Fe(ii) ion by forming Fe(ii)–N bonds.^22–24,33^ As for the Fe(ii)–S codoped g-C_3_N_4_, a S 2p peak located at 163.1 eV can be reasonably assigned to C–S bonds formed in g-C_3_N_4_ lattice *via* substituting N.^34,35^ The peak at 168.0 eV is ascribed to S=O in the intermediate product of sulfoxide resulting from the decomposition of TCA.^20^ By removing the adventitious carbon contamination, the C/N atomic ratio is 0.76 for the pure g-C_3_N_4_, which is fairly close to the stoichiometric value of g-C_3_N_4_. The C/N atomic ratio for the Fe(ii), S codoped g-C_3_N_4_ is about 0.81, which is slightly larger than that of the pure g-C_3_N_4_. XPS results showed that the Fe(ii) and S atoms are doped into g-C_3_N_4_ lattice and may preferentially substitute N atoms. For the Fe(ii)–S codoped g-C_3_N_4_, the S content of atomic percentage is about 0.09% and the Fe(ii) is about 0.20%. Taken XPS and XRD results into consideration, Fe(ii) ions are suggested to position in the pore centre among three tri-*s*-triazine units by forming Fe(ii)–N bonds with the lone-pair sp^2^ electrons of N atoms.

**Fig. 5:**
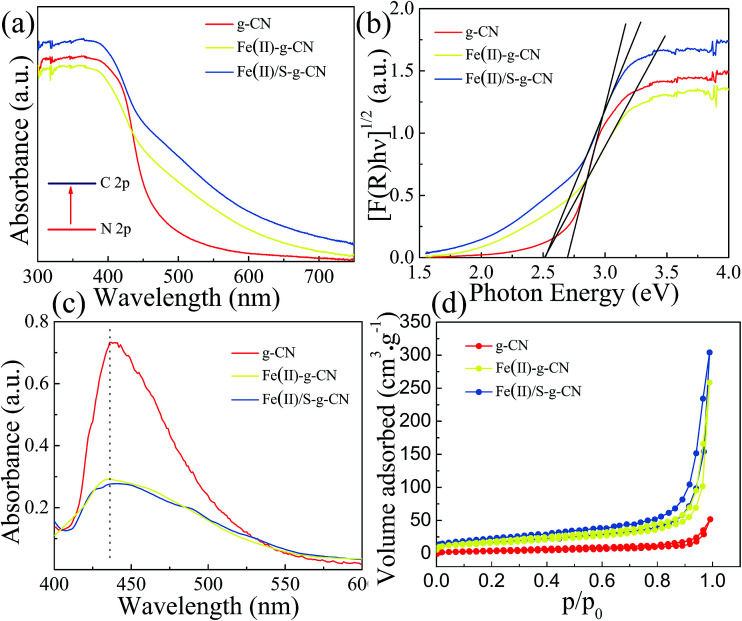
UV-vis absorbance spectra (a), corresponding Kubelka–Munk plots (b), PL (c) and adsorption–desorption isothermal curve of N_2_ (d) of the pure g-C_3_N_4_ nanosheets and Fe(ii)–g-C_3_N_4_ and Fe(ii)/S–g-C_3_N_4_.

The UV-vis diffuses absorbance spectra of the pure, Fe(ii) doped and Fe(ii)–S codoped g-C_3_N_4_ are shown in Fig. 5(a). There is a sharp absorption edge for the pure g-C_3_N_4_ nanosheets at around 460 nm indexing to the bandgap energy of about 2.7 eV, which is associated with the photocatalytic property in visible light.^4,24^ It shows enhanced visible light absorption intensity and the red shift edge *via* Fe(ii) and S doping. The Kubelka–Munk plots in Fig. 4(b) show that the adsorption edge is red shifted with lower bonding energy of 2.51 eV for the Fe(ii) and Fe(ii)–S codoped g-C_3_N_4_. The absorption intensity is remarkably enhanced in the visible region after the Fe(ii) and S doping.^29^ SEM images show that Fe(ii) and S doping causes the g-C_3_N_4_ sheets curling up (Fig. 1(b)). The crimped structure facilitates the n–π* transitions. These results reveal that the Fe(ii)–S doped g-C_3_N_4_ composites could significantly promote the optical absorption performance and enhance the utilization efficiency of solar light, which subsequently results in a higher photocatalytic activity.^28^

The photoluminescence (PL) studies were carried out to investigate the recombination/separation of photoinduced charge carriers in the pure, Fe(ii) doped and Fe(ii)–S codoped g-C_3_N_4_ under the excitation wavelength of 274 nm. The measured PL spectra, as shown in Fig. 5(c), show that all of the samples exhibit a main emission peak appearing at about 440 nm, which is consistent with the reported value in the literature.^34,35^ Compared to the pure g-C_3_N_4_, the Fe(ii) doped and the Fe(ii)–S doped g-C_3_N_4_ give weaker PL intensities revealing the lower recombination probability of photoinduced electrons and holes, which could give rise to a higher photocatalytic activity.

The Fe(ii) and S codoped g-C_3_N_4_ exhibits a largest specific surface area among these three samples, showed in Fig. 5(d). The specific surface area is 13.40 m^2^ g^−1^, 57.28 m^2^ g^−1^, and 59.37 m^2^ g^−1^, and the pore area is 15.48 m^2^ g^−1^, 53.53 m^2^ g^−1^, and 75.84 m^2^ g^−1^ for the pure, Fe(ii) doped, and Fe(ii) + S doped g-C_3_N_4_ catalysts, respectively. Fig. 5(d) indicates that the apparent curves follow the type IV adsorption–desorption isotherm with N_2_ hysteresis loop, which is mainly arisen from the massive presence of mesopores. Therefore, Fe(ii) + S codoped photocatalyst will provide more active sites, facilitating photocatalytic activity.^36,37^

**Fig. 6:**
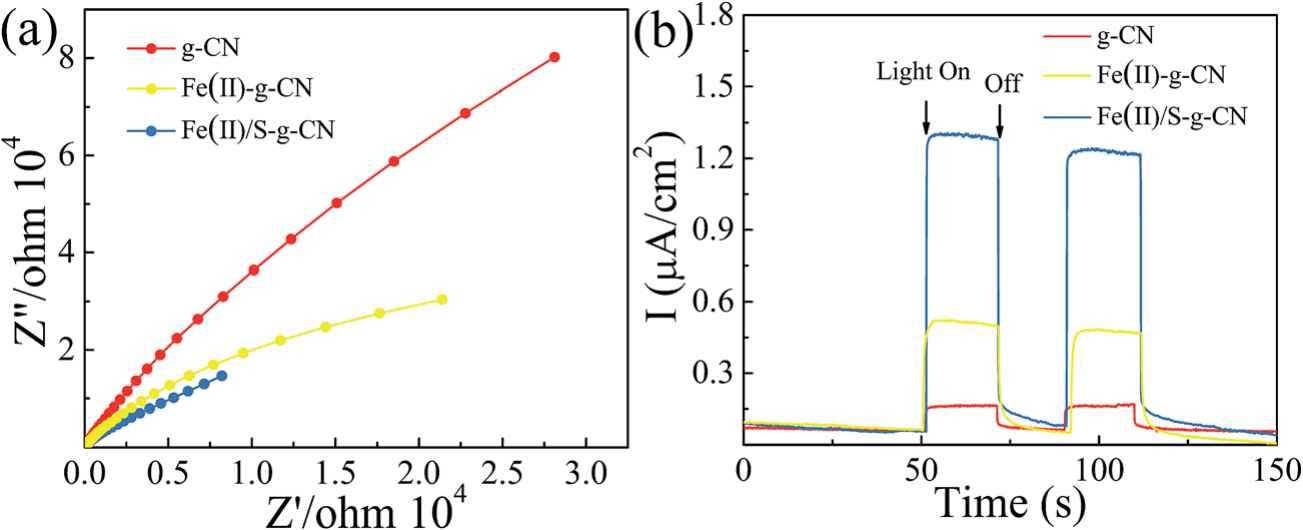
EIS spectra (a), transient photocurrent response (b) of the pure, Fe(ii)–g-C_3_N_4_ and Fe(ii)/S–g-C_3_N_4_ catalysts.

The electrochemical impedance spectra (EIS) of the pure g-C_3_N_4_ and the g-C_3_N_4_ doped with Fe(ii) and S were measured to understand the photocatalytic mechanism. The arc on the EIS Nyquist plot indicates the charge transfer resistance. Generally speaking, the smaller arc radius implies a lower charge transfer resistance.^21,24^ As shown in Fig. 6(a), the Nyquist plots of all the Fe(ii) and S doped g-C_3_N_4_ samples give a smaller arc radius attributing to the reduced electric resistance and enhanced conductivity by doping with Fe(ii) and S elements compared to the pure g-C_3_N_4_. In fact, the arc radius for Fe(ii)–S codoped g-C_3_N_4_ is smallest in all three samples, which is associated with the highest efficiency of the charge separation.^38–41^ Moreover, this changing trend in the arc radius for g-C_3_N_4_ samples is consistent with the results displayed in PL spectra.

The transient photocurrent responses of all samples were recorded in Fig. 6(b). The photocurrent shows a fast response to light irradiation with a good reproducibility for each on–off cycle. That showed a rapid and steady photocurrent response with reproducibility for each on–off cycle. The current density for Fe(ii)–S codoped g-C_3_N_4_ is about 12 times of that observed for pure sample, revealing the most effective separation and transition of photoinduced electron/hole pairs, which is consistent with PL spectra.

**Fig. 7:**
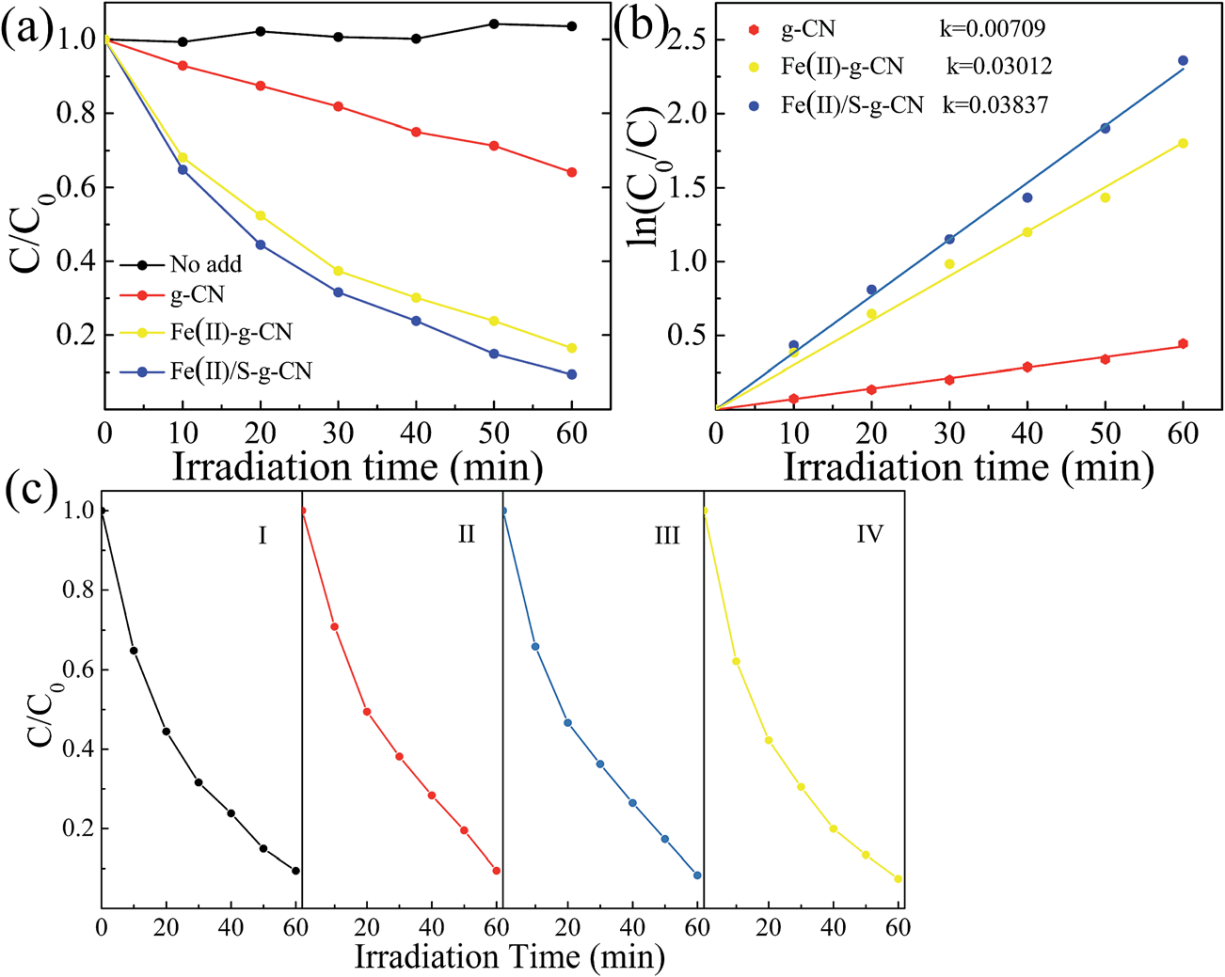
Photocatalytic performances (a) and corresponding first-order reaction kinetics (b) in the degradation of RhB under visible light irradiation for the pure g-C_3_N_4_, the Fe(ii)–g-C_3_N_4_ and Fe(ii)/S–g-C_3_N_4_. Stability test of the Fe(ii)/S–g-C_3_N_4_ (c).

The photocatalytic performances of the pure, Fe(ii) doped and Fe(ii)–S doped g-C_3_N_4_ were evaluated by RhB degradation under visible light irradiation (*λ* > 420 nm), as shown in Fig. 7(a). After 60 min irradiation with visible light, about 83.5% of RhB is degraded in presence of the Fe(ii) doped g-C_3_N_4_, compared to that of only 33.3% of RhB decomposition with the pure g-C_3_N_4_. As for the Fe(ii)–S codoped g-C_3_N_4_, approximately 91% of RhB is decomposed after 60 min with visible light irradiation. There is almost no self-degradation of RhB under visible light irradiation in our experiments. The photodegradation of RhB follows the first order dynamics equation:−ln(*C*_0_/*C*_*t*_) = *kt*where *C*_0_, *C*_*t*_, *k* and *t* are the initial concentration, the concentration at time *t*, reaction coefficient and time *t*, respectively. The first order kinetics, illustrated in Fig. 7(b), shows that the degradation rates for the Fe and Fe/S doped g-C_3_N_4_ are enhanced by 5.4 and 4.2 times compared with the pure material. The stability for the Fe/S doped g-C_3_N_4_ is illustrated in Fig. 7(c). The photodegradation rate does not experience any significant changes after 4 cycles of degradation experiments.

**Fig. 8:**
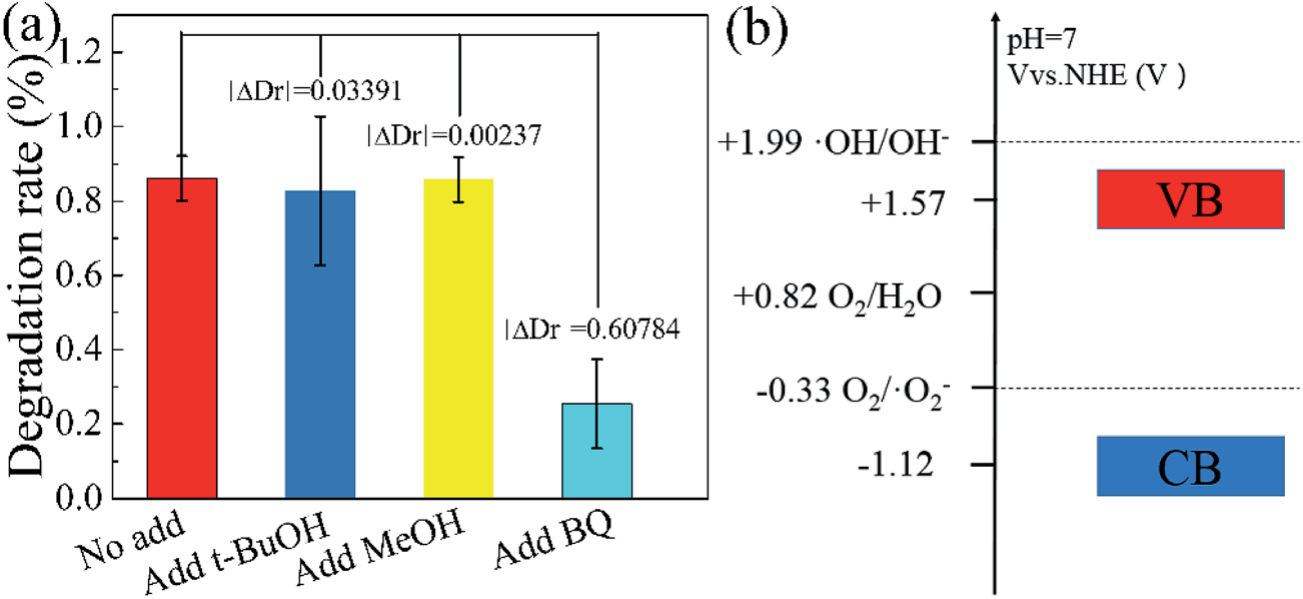
Influence of various scavengers on the visible-light photocatalytic activity of Fe(ii)/S–g-C_3_N_4_ toward the degradation of RhB (a), electric potential diagram of g-C_3_N_4_ and related perssad (b).

In order to further explain the photocatalytic mechanism of the degradation RhB under visible light, benzoquinone (BQ, 1.5 ml, 0.1 mM l^−1^), *tert*-butyl alcohol (*t*-BuOH, 1.5 ml) and methanol (MeOH, 1.5 ml) were added into our model photocatalysis system to trap radical superoxide (˙O_2_^−^), hydroxyl radicals (˙OH) and hole (h^+^),^41,42^ respectively. The degradation rate did not change obviously by adding *t*-BuOH and MeOH in the photocatalytic process, as shown in Fig. 8, indicating hydroxyl radicals and holes are not the reactants for degradating RhB. While the degradation rate decreases dramatically upon adding a small amount of BQ, which clearly indicates that radical superoxide is the primary reactant in the RhB degradation process. The redox potentials for OH/OH^−^ and O_2_/˙O_2_^−^ were determined to be +1.99 V and −0.33 V,^43,44^ respectively. Theoretically, the conduction band and valence band of g-C_3_N_4_ were −1.22 V and +1.57 V.^31,32^ The conduction band of g-C_3_N_4_ is more negative than the redox potential of O_2_/˙O_2_^−^, thus O_2_ can be reduced to O_2_^−^. The valence band of g-C_3_N_4_ is less positive enough to oxide OH^−^ to ˙OH. These results suggest that O_2_^−^ takes part in the photocatalytic process, consistent with our experiment.

In order to get deeper insight into its catalytic mechanism and electronic structure, DFT calculations based on the Gaussian 09 software package were performed to model the Fe(ii) and S doped g-C_3_N_4_ (001) layer with hexagonal honeycomb lattice structure containing three tri-*s*-triazine units. DFT B3LYP/6-31G(d) level of theory was used to optimize the geometry of g-C_3_N_4_ with minimum energy as the initial structure. The highest occupied molecular orbital (HOMO) and the lowest unoccupied molecular orbital (LUMO), the surface electrostatic potential, and density of states were calculated based on B3LPY/Lanl2dz level for Fe(ii), B3LPY/6-311g(d) level for C, N, S.

**Fig. 9:**
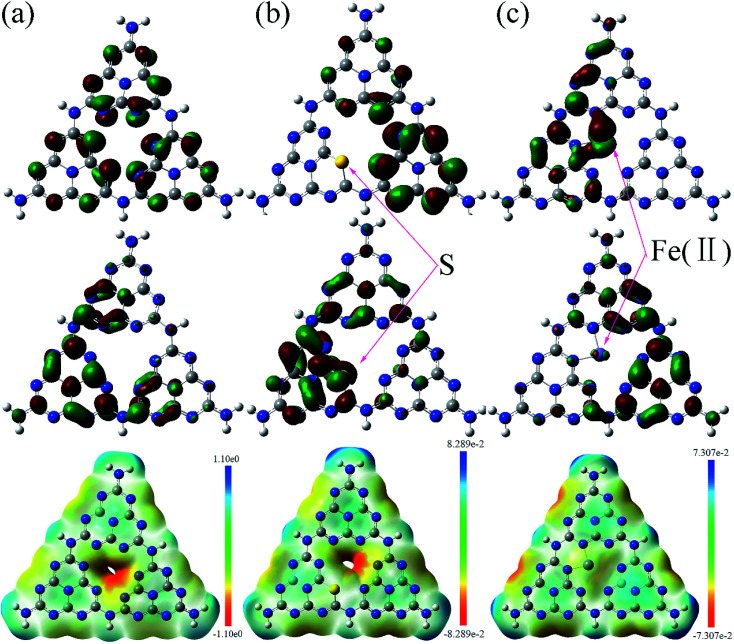
Calculated HOMO (top), LUMO (middle) and surface electrostatic potential (bottom) of the pristine (a), S doped (b) and Fe(ii) doped (c) g-C_3_N_4_ monolayer. The isosurface is taken at a value of 0.003 e bohr^−3^. Carbon atoms are in grey and nitrogen in blue.

Fig. 9 shows the calculated HOMO, LUMO, and the surface electrostatic potential for the (001) lattice plane of g-C_3_N_4_ monolayer. The bigger the separation of HOMO and LUMO, the easier the separation of the photoinduced electron/hole and less the combination of charged carriers. For the pure g-C_3_N_4_ catalyst, the small separation of HOMO and LUMO, as shown in Fig. 9(a) may promote the photoinduced electron/hole recombination, leading to a lower photocatalytic activity. The migration of the photogenerated e^−^/h^+^ pairs is not efficient due to the different localized HOMO and LUMO. HOMO suggests that the edge N atoms provide the oxidation sites for water to O_2_, whereas LUMO indicates that the edge C and the inner N atoms are the preferred reduction sites to form H_2_.^45,46^ There are no HOMO and LUMO at the bridge N atoms inhibiting the carrier migration from one heptazine unit to another, reducing the photocatalytic performance. Fig. 9(b) shows the calculated molecular orbitals of S doped g-C_3_N_4_, which demonstrates that the separation of HOMO and LUMO increases distinctly with the sulfur atom doping. The S atoms act as the reduction sites. The substitution of the edge N with S causes slightly stronger delocalized HOMO and LUMO compared to the pure g-C_3_N_4_ monolayer. The dispersion of the HOMO and LUMO distribution can enhance the carrier mobility. The substitution of the edge N with S causes stronger delocalized HOMO and LUMO, thus increases the reactive sites. Fig. 9(c) presents the calculated molecular orbitals of the Fe(ii) doped g-C_3_N_4_. The separation of HOMO and LUMO increases after Fe(ii) ion is imbedded in the pore centre among the three triazine units in the layered molecules. These results indicate that the separation of HOMO and LUMO increases with Fe(ii) and S doping with S doping altering the electronic structure of triazine unit and Fe(ii) doping enhancing the density of states of the pore among three triazine units. Our experimental study showed that ˙O_2_^−^ is the main active species in the photodegradation of RhB. Thus, the surface electrostatic potential of the photocatalysts was calculated to investigate the separation of ˙O_2_^−^ at the LUMO energy band from the catalyst surface, as shown at the bottom in Fig. 9. It is shown that the pore site has a negative potential, thus is the optimum site for ˙O_2_^−^ separating from the surface of g-C_3_N_4_. The potentials deceases further with the Fe(ii) and S doping, facilitating the separation of ˙O_2_^−^ and enhancing the photocatalytic activity.

**Fig. 10:**
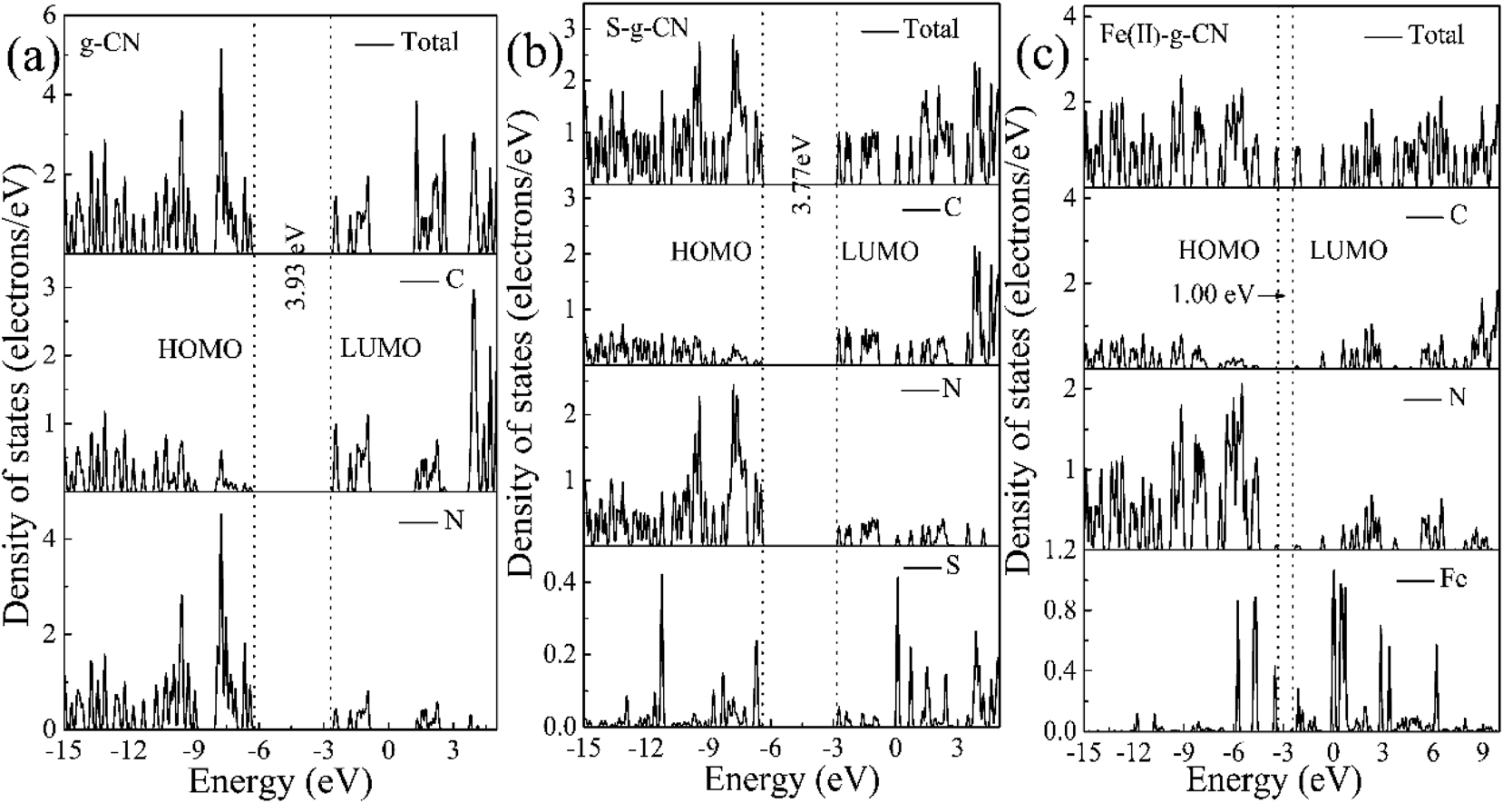
Calculated total and partial DOS plots of C, N, S and Fe(ii) elements for the pristine (a), S doped (b) and (c) Fe(ii) doped g-C_3_N_4_ monolayer.

The total and partial density of states (DOS) for the pure g-C_3_N_4_ is shown in Fig. 10(a). The DOS in valance band is mainly contributed by the nitrogen atoms, while the DOS in conduction band is from the carbon atoms. The bandgap is narrowing upon the S and Fe(ii) doping and the Fermi level is shifted towards the conduction band, as shown in Fig. 10(b and c). The S doping changes the electronic structure of g-C_3_N_4_ by contributing to both valence and conduction bands. The bandgap between HOMO and LUMO is 3.77 eV for the S doped g-C_3_N_4_. Fe(ii) doping in the lattice of g-C_3_N_4_ generates discrete energy level in the bandgap, thus deceasing the bandgap significantly to 1.0 eV compared to the pure g-C_3_N_4_ with a bandgap of 3.93 eV. Therefore, our DFT calculation indicates that the nonmetal element S mainly changes the electronic structure of triazine unit, while the metal Fe(ii) ion imbedded in the pore alters the energy band structure by exerting discrete energy level in the bandgap.^47,48^ The valence electron divergence between the dopant atoms and the adjacent intrinsic atoms yields new energy band in the g-C_3_N_4_ monolayer, thus changes its photocatalytic performance.

**Scheme 1:**
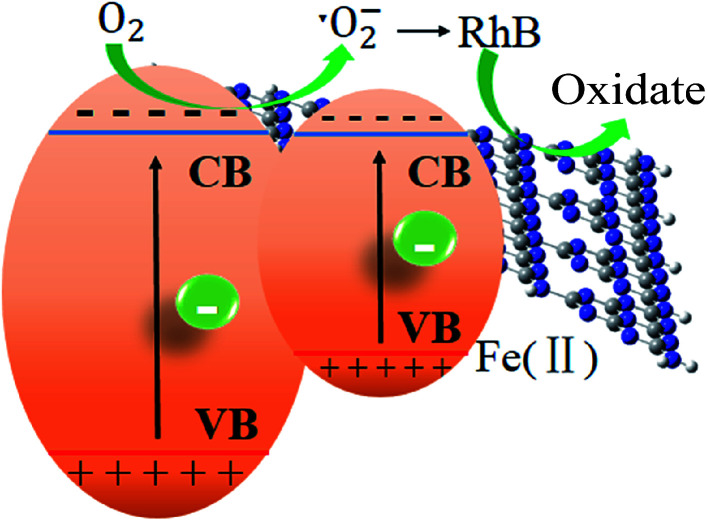
Z-scheme photocatalytic systems.

Finally, a tentative mechanism for photocatalytic degradation of RhB was proposed by taking above-mentioned experimental and theoretical strands into consideration. The photocatalytic mechanism of the degradation of RhB was illustrated in Scheme 1. S doping alters the electronic structure of triazine unit, while Fe ion forms a new impurity band above the valence band of the pure g-C_3_N_4_. This impurity band improves the separation of photoinduced electron/hole pairs. The photoinduced electrons jump more easily to the conduction band of g-C_3_N_4_ for the Fe impurity band locates above the valence band acting a bridge for electron transfer. The photoexcited electrons generated from g-C_3_N_4_ under visible-light irradiation would jump into the conduction band and combine with the dissolved O_2_ to form ˙O_2_^−^, with which RhB molecules were decomposed. The doping of Fe^2+^ significantly lowers the bandgap, enabling the harvest of major visible light and generating photoinduced electrons in the conduction band of g-C_3_N_4_, leading to a significant enhancement of the photocatalytic activity.

## 4. Conclusion

In summary, the Fe(ii) and S codoped g-C_3_N_4_ photocatalyst was successfully prepared by a thermal condensation process of melamine, ferrous chloride and trithiocyanuric acid. Compared to the pure g-C_3_N_4_ nanosheets, the photocatalytic performance for RhB degradation is enhanced by a factor of 5.4 times for the Fe(ii)–S codoped g-C_3_N_4_, in which Fe(ii) coordinates in the pore centre by forming Fe(ii)–N bonds and S atoms substitute the N atoms in the triazine unit. The stronger delocalization of HOMO and LUMO together with bandgap narrowing by Fe(ii) and S codoping facilitates the photoexcitation and migration of photoinduced charge carriers, thus enhancing the photocatalytic performance. The experimental and theoretical results confirm that the band structure of g-C_3_N_4_ could be tuned *via* Fe(ii) + S doping, thus improving the photocatalytic activity.

## Author contributions

The manuscript was written through contributions of all authors. All authors have given approval to the final version of the manuscript.

## Conflicts of interest

There are no conflicts to declare.

## Supplementary Material

## References

[cit1] Fujishima A., Honda K. (1972). Electrochemical photolysis of water at a semiconductor electrode. Nature.

[cit2] Wang X., Maeda K., Thomas A., Takanabe K., Xin G., Carlsson J. M., Domen K., Antonietti M. (2009). A metal-free polymeric photocatalyst for hydrogen production from water under visible light. Nat. Mater..

[cit3] Kroke E., Schwarz M., Horath-Bordon E., Kroll P., Noll B., Norman A. D. (2002). Tri-*s*-triazine derivatives. Part I: From trichloro-tri-*s*-triazine to graphitic C_3_N_4_ structures part II: Alkalicyamelurates M_3_[C_6_N_7_O_3_], M = Li, Na, K, Rb, Cs. New J. Chem..

[cit4] Ong W. J., Tan L. L., Ng Y. H., Yong S. T., Chai S. P. (2016). Graphitic carbon nitride (g-C_3_N_4_)-based photocatalysts for artificial photosynthesis and environmental remediation: are we a step closer to achieving sustainability?. Chem. Rev..

[cit5] Martín-Ramos P., Martín-Gil J., Dante R. C., Vaquero F., Navarro R. M., Fierro J. L. G. (2015). A simple approach to synthesize g-C_3_N_4_ with high visible light photoactivity for hydrogen production. Int. J. Hydrogen Energy.

[cit6] Shiraishi Y., Kanazawa S., Sugano Y., Tsukamoto D., Sakamoto H., Ichikawa S., Hirai T. (2014). Highly selective production of hydrogen peroxide on graphitic carbon nitride (g-C_3_N_4_) photocatalyst activated by visible light. ACS Catal..

[cit7] Cui Y., Ding Z., Liu P., Antonietti M., Fu X., Wang X. (2012). Metal-free activation of H_2_O_2_ by g-C_3_N_4_ under visible light irradiation for the degradation of organic pollutants. Phys. Chem. Chem. Phys..

[cit8] Shi H., Chen G., Zhang C., Zou Z. (2014). Polymeric g-C_3_N_4_ Coupled with NaNbO_3_ nanowires toward enhanced photocatalytic reduction of CO_2_ into renewable fuel. ACS Catal..

[cit9] Zhao Z., Sun Y., Dong F. (2015). Graphitic carbon nitride based nanocomposites: a review. Nanoscale.

[cit10] Wen J., Xie J., Chen X., Li X. (2017). A review on g-C_3_N_4_-based photocatalysts. Appl. Surf. Sci..

[cit11] Gao J., Miao J., Li P. Z., Teng W. Y., Yang L., Zhao Y., Liu B., Zhang Q. (2014). A p-type Ti(iv)-based metal-organic framework with visible-light photo-response. Chem. Commun..

[cit12] Wang J. P., Cong J., Xu H., Wang J. M., Liu H., Liang M., Gao J., Ni Q., Yao J. (2017). Facile gel-based morphological control of Ag/g-C_3_N_4_ porous nanofibers for photocatalytic hydrogen generation. ACS Sustainable Chem. Eng..

[cit13] Yan S. C., Li Z. S., Zou Z. G. (2010). Photodegradation of rhodamine B and methyl orange over boron-doped g-C_3_N_4_ under visible light irradiation. Langmuir.

[cit14] Li J., Shen B., Hong Z., Lin B., Gao B., Chen Y. (2012). A facile approach to synthesize novel oxygen-doped g-C_3_N_4_ with superior visible-light photoreactivity. Chem. Commun..

[cit15] Zhou Y., Zhang L., Liu J., Fan X., Wang B., Wang M., Ren W., Wang J., Li M., Shi J. (2015). Brand new P-doped g-C_3_N_4_: enhanced photocatalytic activity for H_2_ evolution and rhodamine B degradation under visible
light. J. Mater. Chem. A.

[cit16] Ge L., Han C., Xiao X., Guo L., Li Y. (2013). Enhanced visible light photocatalytic hydrogen evolution of sulfur-doped polymeric g-C_3_N_4_ photocatalysts. Mater. Res. Bull..

[cit17] Hu S., Jin R., Lu G., Liu D., Gui J. (2014). The properties and photocatalytic performance comparison of Fe^3+^-doped g-C_3_N_4_ and Fe_2_O_3_/g-C_3_N_4_ composite catalysts. RSC Adv..

[cit18] Fan M., Song C., Chen T., Yan X., Xu D., Gu W., Shi W., Xiao L. (2016). Visible-light-drived high photocatalytic activities of Cu/g-C_3_N_4_ photocatalysts for hydrogen production. RSC Adv..

[cit19] Chen L., Man Y., Chen Z., Zhang Y. (2016). Ag/g-C_3_N_4_ layered composites with enhanced visible light photocatalytic performance. Mater. Res. Express.

[cit20] Xue J., Ma S., Zhou Y., Zhang Z., He M. (2015). Facile photochemical synthesis of Au/Pt/g-C_3_N_4_ with plasmon-enhanced photocatalytic activity for antibiotic degradation. ACS Appl. Mater. Interfaces.

[cit21] Kumar S., Baruah A., Tonda S., Kumar B., Shanker V., Sreedhar B. (2014). Cost-effective and eco-friendly synthesis of novel and stable N-doped ZnO/g-C_3_N_4_ core-shell nanoplates with excellent visible-light responsive photocatalysis. Nanoscale.

[cit22] Yan H., Yang H. (2011). TiO_2_–g-C_3_N_4_ composite materials for photocatalytic H_2_ evolution under visible light irradiation. J. Alloys Compd..

[cit23] Zhang J., Wang Y., Jin J., Zhang J., Lin Z., Huang F., Yu J. (2013). Efficient visible-light photocatalytic hydrogen evolution and enhanced photostability of core/shell CdS/g-C_3_N_4_ nanowires. ACS Appl. Mater. Interfaces.

[cit24] Wang J., Xu H., Qian X., Dong Y., Gao J., Qian G., Yao J. (2015). Direct synthesis of porous nanorod-type graphitic carbon nitride/CuO composite from Cu-melamine supramolecular framework towards enhanced photocatalytic performance. Chem.–Asian J..

[cit25] You R., Dou H., Chen L., Zheng S., Zhang Y. (2017). Graphitic carbon nitride with S and O codoping for enhanced visible light photocatalytic performance. RSC Adv..

[cit26] Gao J., Wang Y., Zhou S., Lin W., Kong Y. (2017). A Facile One-Step Synthesis of Fe-Doped g-C_3_N_4_ Nanosheets and Their Improved Visible-Light Photocatalytic Performance. ChemCatChem.

[cit27] Li X., Zhang J., Shen L., Ma Y., Lei W., Cui Q., Zou G. (2008). Preparation and characterization of graphitic carbon nitride through pyrolysis of melamine. Appl. Phys. A.

[cit28] Liu Q., Guo Y., Chen Z., Zhang Z., Fang X. (2016). Constructing a novel ternary Fe(iii)/graphene/g-C_3_N_4_ composite photocatalyst with enhanced visible-light driven photocatalytic activity *via* interfacial charge transfer effect. Appl. Catal., B.

[cit29] Yang Y., Guo Y., Liu F., Yuan X., Guo Y., Zhang S., Guo W., Huo M. (2013). Preparation and enhanced visible-light photocatalytic activity of silver deposited graphitic carbon nitride plasmonic photocatalyst. Appl. Catal., B.

[cit30] Ge L., Han C. (2012). Synthesis of MWNTs/g-C_3_N_4_ composite photocatalysts with efficient visible light photocatalytic hydrogen evolution activity. Appl. Catal., B.

[cit31] Qi Y., Wu W., Han L., Qu H., Han X., Wang A., Xu J. (2015). Using TG-FTIR and XPS to understand thermal degradation and flame-retardant mechanism of flexible poly(vinyl chloride) filled with metallic ferrites. J. Therm. Anal. Calorim..

[cit32] Zhang X., Xie X., Wang H., Zhang J., Pan B., Xie Y. (2013). Enhanced photoresponsive ultrathin graphitic-phase C_3_N_4_ nanosheets for bioimaging. J. Am. Chem. Soc..

[cit33] Zhang S., Li J., Zeng M., Li J., Xu J., Wang X. (2014). Bandgap engineering and mechanism study of nonmetal and metal ion codoped carbon nitride: C + Fe as an example. Chem.–Eur. J..

[cit34] Lindberg B., Hamrin K., Kloster-Jensen E., Stølevik R., Werner P. (1970). Substituent Effects of Sulfur Groups III. The Influence of Conjugation on ESCA Spectra of Sulfur Substituted Nitrobenzenes. Acta Chem. Scand..

[cit35] Lindberg B. J., Hamrin K., Johansson G., Gelius U., Fahlman A., Nordling C., Siegbahn K. (1970). Molecular spectroscopy by means of ESCA II. Sulfur compounds. Correlation of electron binding energy with structure. Phys. Scr..

[cit36] Miranda C., Mansilla H., Yáñez J., Obregón S., Colón G. (2013). Improved photocatalytic activity of g-C_3_N_4_/TiO_2_ composites prepared by a simple impregnation method. J. Photochem. Photobiol., A.

[cit37] He F., Chen G., Zhou Y., Yu Y., Zheng Y., Hao S. (2015). Facile synthesis of mesoporous g-C_3_N_4_ with highly enhanced photocatalytic H_2_ evolution performance. Chem. Commun..

[cit38] Li Z., Kong C., Lu G. (2015). Visible photocatalytic water splitting and photocatalytic two-electron oxygen formation over Cu- and Fe-doped g-C_3_N_4_. J. Phys. Chem. C.

[cit39] Kong H. J., Won D. H., Kim J., Woo S. I. (2016). Sulfur-doped g-C_3_N_4_/BiVO_4_ composite photocatalyst for water oxidation under visible light. Chem. Mater..

[cit40] Wang K., Li Q., Liu B., Cheng B., Ho W., Yu J. (2015). Sulfur-doped g-C_3_N_4_ with enhanced photocatalytic CO_2_-reduction performance. Appl. Catal., B.

[cit41] Kou J., Li Z., Yuan Y., Zhang H., Wang Y., Zou Z. (2009). Visible-light-induced photocatalytic oxidation of polycyclic aromatic hydrocarbons over tantalum oxynitride photocatalysts. Environ. Sci. Technol..

[cit42] Yamakata A., Ishibashi A. T., Onishi H. (2002). Electron- and hole-capture reactions on Pt/TiO_2_ photocatalyst exposed to methanol vapor studied with time-resolved infrared absorption spectroscopy. J. Phys. Chem. B.

[cit43] Mrowetz M., Balcerski W., Colussi A. J., Hoffmann M. R. (2004). Oxidative power of nitrogen-doped TiO_2_ photocatalysts under visible illumination. J. Phys. Chem. B.

[cit44] Liu G., Niu P., Yin L., Cheng H. M. (2012). Alpha-sulfur crystals as a visible-light-active photocatalyst. J. Am. Chem. Soc..

[cit45] Cui J., Liang S., Wang X., Zhang J. (2015). First principle modeling of oxygen-doped monolayer graphitic carbon nitride. Mater. Chem. Phys..

[cit46] Zheng Q., Durkin D. P., Elenewski J. E., Sun Y., Banek N. A., Hua L., Chen H., Wagner M. J., Zhang W., Shuai D. (2016). Visible-light-responsive graphitic carbon nitride: rational design and photocatalytic applications for water treatment. Environ. Sci. Technol..

[cit47] Stolbov S., Zuluaga S. (2013). Sulfur doping effects on the electronic and geometric structures of graphitic carbon nitride photocatalyst: insights from first principles. J. Phys.: Condens. Matter.

[cit48] Oh Y., Hwang J. O., Lee E. S., Yoon M., Le V. D., Kim Y. H., Kim D. H., Kim S. O. (2016). Divalent Fe atom coordination in two-dimensional microporous graphitic carbon nitride. ACS Appl. Mater. Interfaces.

